# Editorial: New epidemiological, etiological and management insights into community-acquired pneumonia in children: subregional, regional and global perspectives

**DOI:** 10.3389/fmed.2023.1275263

**Published:** 2023-09-20

**Authors:** Adegoke G. Falade, Richard A. Adegbola

**Affiliations:** ^1^Department of Paediatrics, University of Ibadan, and University College Hospital, Ibadan, Nigeria; ^2^Nigerian Institute of Medical Research (NIMR), Lagos, Nigeria

**Keywords:** community-acquired pneumonia, antibiotic guidelines, mycoplasma pneumoniae mucus plug, antibiotic stewardship, under-5

Community-acquired pneumonia (CAP) is one of the most common causes of under-5 mortalities in resource-constrained countries, especially in sub-Saharan and Southeast Asian countries (1), despite the efforts of the World Health Organization (WHO) and UNICEF through the Integrated Global Action Plan for the Prevention and Control of Pneumonia and Diarrhea (2). Consequently, there is an urgent need for evidence-based and sustainable approaches to target childhood pneumonia. The aim of this collection is to bring together the latest quality articles from researchers working in the area of community-acquired pneumonia in children. In this Research Topic, we pulled together three original research articles, and one hypothesis and theory article, to give an overview of the status in this field.

Antibiotics in the treatment of severe CAP in low-resource settings are primarily focused on *Streptococcus pneumoniae* and *Haemophilus influenzae* type b (Hib), which are the two most common etiologic agents. In a few cases, anti-staphylococcal antibiotics are given for *Staphylococcus aureus*. Recently, infants are being vaccinated with pneumococcal and Hib conjugate vaccines with increasing coverage. Consequently, the most common etiologic agents of pneumonia are viral. Nevertheless, antibiotics are still being given to all cases of severe CAP because there is no simple way to distinguish between viral pneumonia and bacterial pneumonia. It is important to observe antibiotic stewardship to lessen the development of antimicrobial resistance. In light of this, Farida et al. have developed a multivariable prediction model on data from a multisite observational clinical and etiological cohort study in Indonesia, which helps clinicians treating severe CAP to select likely bacterial pneumonia for appropriate and effective antibiotics. However, its replicability and predictive accuracy of bacterial etiology remain to be studied in CAP patients from other low-income countries.

Antibiotic guidelines in the treatment of severe CAP in under-5s have been developed. The parenteral combination of ampicillin or benzylpenicillin and gentamicin is the first line, while parenteral ceftriaxone is the second line. However, many clinicians tend to use ceftriaxone because of the belief that it is a wide-spectrum antibiotic and, therefore, should be more effective than the first line. The first evidence-based study came from the work of La Vecchia et al. on children aged 2–59 months in hospitals in southern Ethiopia, an east African country, which showed that the WHO first-line recommended treatment for severe pneumonia is not inferior to the use of ceftriaxone, which should be used only as a second-line treatment.

Pneumonia deaths are predominantly at the community, primary healthcare, and secondary healthcare levels. Community prevention and treatment of pneumonia have been highlighted as very important in the reduction of pneumonia burden. The first step in this regard is the care-seeking practice for childhood pneumonia among caregivers. Bakare et al. conducted a cross-sectional household survey on eligible women and their under-5 children in Kiyawa Local Government Area, Jigawa State, Nigeria, as part of the baseline phase of the Jigawa community-based cluster-randomized controlled trial. A major finding is that caregiver's knowledge of the disease was not associated with care seeking for children with symptoms of Acute Respiratory Infection. Therefore, interventions focused on the reduction of child mortality should be more robust than solely giving information on symptoms and risk factors of pneumonia to caregivers.

Infection with *Mycoplasma pneumoniae* (MP) mostly occurs in an outpatient setting. Sometimes, it may be severe, requiring hospitalization, especially among the elderly population and immunocompromised patients. One of the complications of severe MP pneumonia in children is airway blockage by mucus plugs, which may lead to chronic lung infection. Vitamin D is an immunomodulator, and it imparts the innate and adaptive immunity of the respiratory system positively. In their hypothesis and theory article, Kun et al. investigated associations between serum 25(OH)D levels and mucus plugs complicating *M. pneumoniae* pneumonia. Low levels of serum 25(OH)D are associated with the formation of mucus plugs in the airway. Hence, this can be used as a test for the presence of mucus plugs in children suffering from severe MP pneumonia.

## Author contributions

AF: Writing—original draft, Writing—review and editing. RA: Writing—original draft, Writing—review and editing.
